# Supination adduction stage 2 associated with transverse fracture of the lateral malleolus and rupture of the anterior talofibular ligament: a case report and literature review

**DOI:** 10.3389/fsurg.2025.1658026

**Published:** 2025-09-22

**Authors:** Jingxuan Wang, Kangyong Yang, Zhenjiang Liu, Ke Jie, Biqing Huang, Shiheng Wang, Zhihong Mo, Yunxuan Zou

**Affiliations:** ^1^ The Eighth Clinical Medical College of Guangzhou University of Chinese Medicine, Foshan, Guangdong, China; ^2^ Foshan Hospital of Traditional Chinese Medicine, Foshan, Guangdong, China; ^3^ Wangjing Hospital, China Academy of Chinese Medical Sciences, Beijing, China

**Keywords:** ankle fracture, Lauge-Hansen classification, supination-adduction, anterior talofibular ligament rupture, failure point, case reports

## Abstract

**Background:**

Supination-adduction (SAD) type ankle fractures occur when the ankle is subjected to inversion forces while in a supinated position, leading to transverse fractures of the lateral malleolus or lateral ligament injuries, often accompanied by vertical fractures of the medial malleolus. Unlike the typical SAD pattern, the concurrent occurrence of a transverse lateral malleolus fracture combined with rupture of the Anterior Talofibular Ligament (ATFL) is uncommon and has not been reported in the literature; such injuries are frequently missed in clinical practice, which in turn affects clinicians’ treatment decisions and the recovery of ankle joint stability and function.

**Case presentation:**

This report describes a case of an adult Asian female patient who sustained a left ankle injury due to a missed step, resulting in swelling and pain. The initial diagnosis was a left double ankle fracture (SAD stage 2). During surgery, after stabilizing the medial and lateral malleoli, fluoroscopy revealed that the talus could not be reduced. An extended incision identified the ATFL rupture, which was subsequently repaired using the Broström-Gould technique. Post-repair fluoroscopy confirmed satisfactory reduction of the talus and proper alignment of the ankle joint. After two weeks of cast immobilization, the patient began gradual rehabilitation exercises. At the 18-month follow-up, the patient exhibited good ankle function, achieving an American Orthopaedic Foot and Ankle Society Ankle - Hindfoot Scale of 100.

**Conclusion:**

This report shares the clinical experience in diagnosing and treating occult injuries of the ATFL in a case of SAD stage 2 ankle fracture to enhance awareness and prevent missed diagnoses in similar injuries. We emphasize that in SAD stage 2 fractures showing unexplained talar tilt after fixation, clinicians should suspect and evaluate for occult ATFL injury to avoid missed diagnoses and optimize treatment decisions.

## Introduction

Supination-adduction (SAD) fractures are a common type of ankle fracture. According to the Lauge-Hansen classification, the mechanism of injury is characterized by supination of the ankle joint coupled with adduction force, typically resulting in a transverse fracture of the lateral malleolus or damage to the lateral ligaments, and often accompanied by a vertical fracture of the medial malleolus (1, 2). A thorough understanding of the fracture mechanism is crucial for devising precise treatment strategies.

However, there are variant presentations in clinical practice that differ from the classical description. In this paper, we report a rare variation of a SAD fracture, in which the patient presented with both a transverse fracture of the lateral malleolus and a complete rupture of the Anterior Talofibular Ligament (ATFL), which was not detected preoperatively and intraoperatively proved to be an impediment to talar reduction. Based on this, the aims of this report are to present in detail the clinical manifestations, intraoperative findings and management, and follow-up functional outcomes of this combination of injuries, and to alert clinicians to heighten their vigilance for occult ligamentous injury in cases of SAD stage 2 fractures that develop unexplained talar tilt after fixation, thereby optimizing diagnostic and therapeutic decision-making.

## Patient information

**Figure 1:**
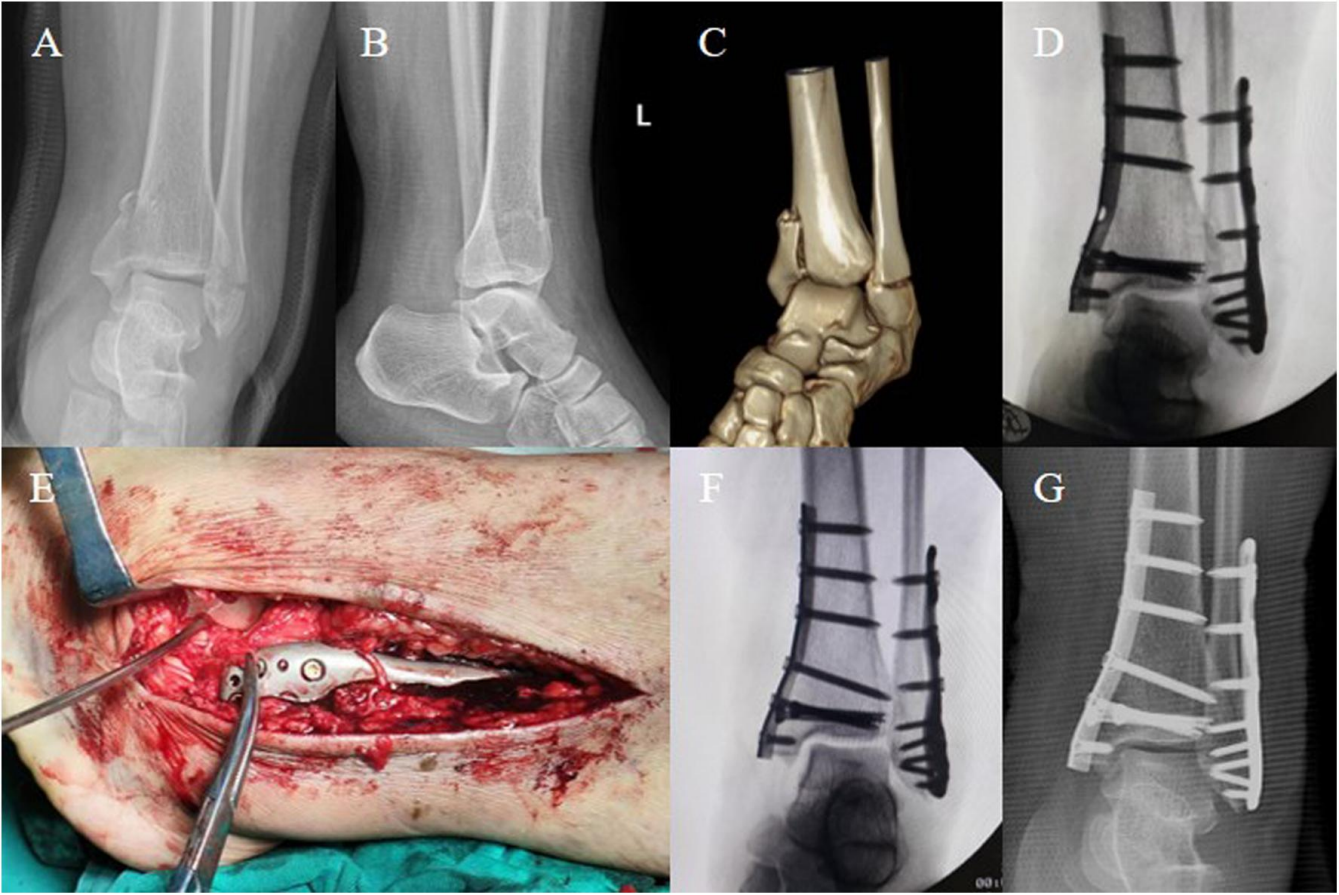
A 31-year-old Asian female (BMI: 30.4 kg/m^2^) sustained a left ankle sprain, and preoperative imaging diagnosed a bimalleolar fracture. **(A–C)** Preoperative DR images and CT scans show fractures of the medial and lateral malleolus in the left ankle, classified as SAD stage 2 according to the Lauge-Hansen classification. **(D)** Intraoperative fluoroscopy after stabilizing the medial and lateral malleoli indicates poor reduction of the talus, with a Talar Tilt angle (TT angle) of 6.5°. **(E)** Extension of the lateral incision revealed rupture of ATFL, with only a small amount of ineffective ligament fibers present. **(F)** The lateral ligaments were repaired using the Broström-Gould technique, and fluoroscopy showed satisfactory reduction of the talus and proper alignment of the ankle joint, with a TT angle of 0.5°. **(G)** Postoperative anteroposterior DR image demonstrates satisfactory positioning of the internal fixation and good alignment of the ankle joint.

A 31-year-old Asian female (BMI: 30.4 kg/m^2^) sustained a left ankle sprain from a misstep two days before presentation, resulting in swelling, pain, and limited mobility. She underwent an examination at a local hospital, where DR images indicated fractures of the left medial and lateral malleoli. Seeking further treatment, she visited our hospital. She has an otherwise healthy medical history. Physical examination revealed grade 3 swelling in the left ankle joint, significant tenderness in both the medial and lateral malleoli, limited joint movement due to pain, normal dorsalis pedis pulse, and good distal toe perfusion. DR and CT images of the ankle showed a transverse fracture of the distal fibula and a vertical fracture of the medial malleolus (Figures 1A–C), consistent with a SAD fracture as per the Lauge-Hansen classification. No neurovascular injuries were found. Preoperative diagnosis was a left bimalleolar fracture, with a plan for surgical intervention.

Under spinal anesthesia, the patient was positioned supine. After routine disinfection and application of a tourniquet on the left lower limb, a medial incision was made at the left ankle to expose the distal tibia, revealing a comminuted fracture of the medial malleolus with bone fragment separation. The distal medial tibial articular surface was cleaned, and the fracture was reduced under direct vision, temporarily fixed with Kirschner wires. Fluoroscopy confirmed satisfactory reduction. A one-third tubular plate (Zhengtian, China) was used for fixation, reinforced with two 4.0-mm cannulated screws (Zhengtian, China). Subsequently, a lateral incision at the left ankle exposed a transverse fracture of the lateral malleolus. After reduction, it was temporarily fixed with Kirschner wires, and then secured with a lateral locking plate (Zhengtian, China) and multiple screws. C-arm fluoroscopy showed appropriate internal fixation, but the talus remained in varus tilt (Figure 1D). The anterior drawer test was positive. Considering the patient's history, physical examination, fracture classification, and intraoperative fluoroscopy, an ATFL injury was suspected. Thus, the incision was extended to explore further, revealing a ruptured ATFL entrapped in the tibiofibular joint space (Figure 1E). Then, a 3.0-mm bone anchor (Arthrex, USA) was placed at the tip of the lateral malleolus, and the ligament and superior extensor retinaculum were sutured using the Broström-Gould technique (to maintain eversion knotting). Fluoroscopy confirmed the correction of talar tilt and restoration of ankle congruence (Figure 1F). The surgical area was irrigated, stepwise sutured, the tourniquet released, and the wound dressed with alcohol gauze and externally stabilized with a plaster cast. The surgery was completed successfully, and the patient remained hemodynamically stable and was safely returned to the ward with adequate distal toe perfusion. Immediate postoperative DR images showed that, as during the surgery, the internal fixation was in a satisfactory position, and the ankle joint alignment was good (Figure 1G).

**Figure 2:**
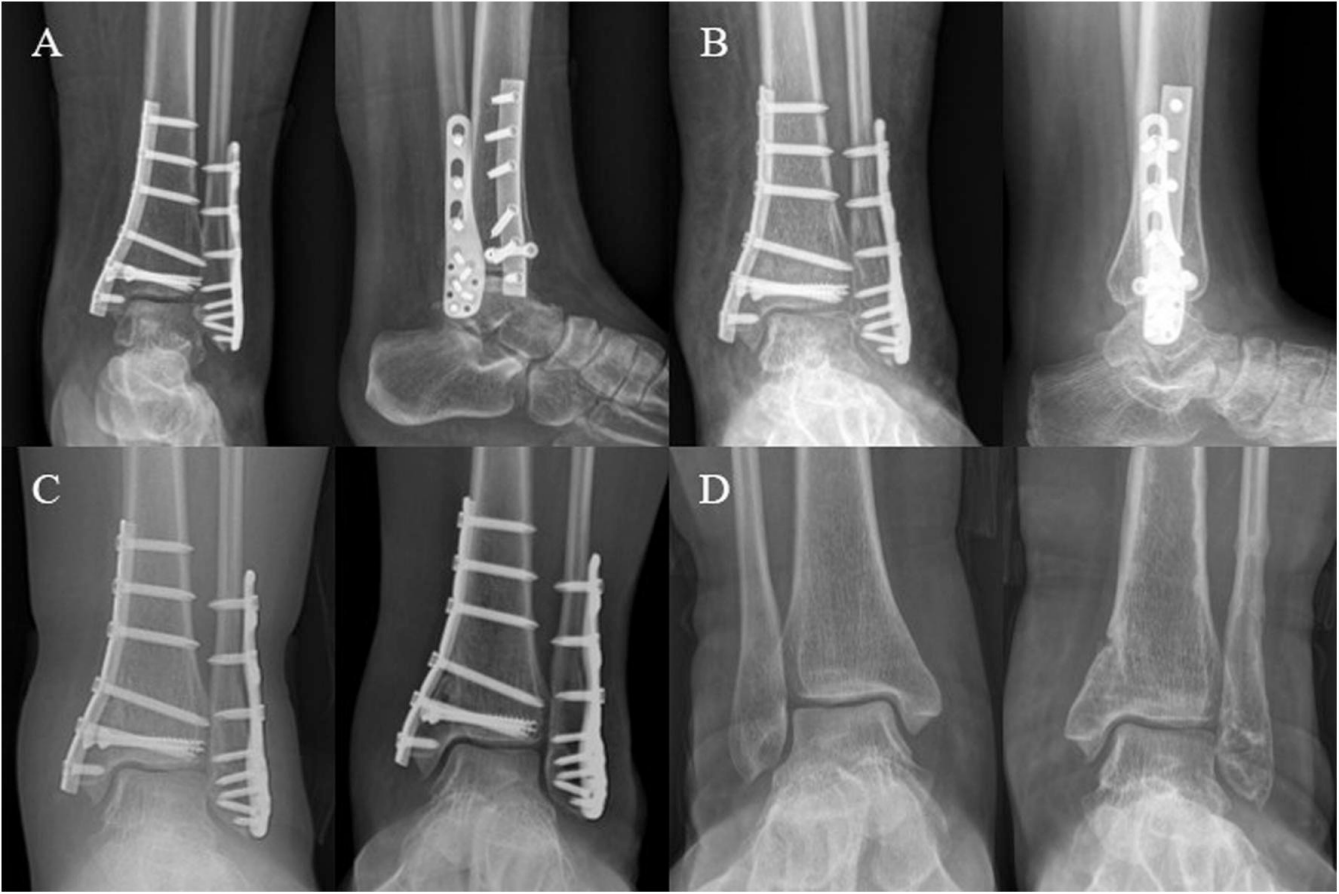
This figure illustrates the postoperative follow-up timeline. **(A,B)** Anteroposterior and lateral DR images at 1 and 3 months post-operation show gradual healing of the ankle fracture. **(C)** Anteroposterior images at 9 months and 1 year post-operation demonstrate satisfactory alignment of the ankle joint. **(D)** At 18 months post-operation, anteroposterior images of both ankles show that after removal of the internal fixation, the alignment of the joint remains good when compared to the contralateral joint.

After surgery, the ankle was immobilized with a short-leg cast for 2 weeks, followed by active and passive non-weight-bearing exercises. At 4 weeks, partial weight-bearing was initiated (Figure 2A). Three months postoperatively, DR images showed resolution of the fracture lines in the medial and lateral malleoli, allowing the patient to progress to full weight-bearing (Figure 2B). At 9 and 13 months postoperative follow-ups, the patient exhibited good ankle function with a negative anterior drawer test, achieving an American Orthopaedic Foot and Ankle Society Ankle-Hindfoot Scale (AOFAS Ankle-Hindfoot Scale) of 100, and during the same period, the plate and all screws were removed (Figure 2C). At the 18-month follow-up, the patient's ankle recovery remained consistent with previous outcomes, proving satisfactory (Figure 2D).

This study was approved by the Medical Ethics Committee of Foshan Hospital of Traditional Chinese Medicine, approval number: [KY (2025) 257]. Written informed consent was obtained from the patient for the information and related images included in this study. This case report adheres strictly to the SCARE and CARE case report guidelines (3, 4).

## Discussion

Ankle fractures are among the most common orthopedic injuries, accounting for 9% of all fractures (5), with an annual incidence of 107–174 per 100,000 people (6–8). Various classification systems have been introduced in clinical practice to assess ankle fractures, including the Lauge-Hansen classification, Danis-Weber classification, and AO/OTA classification, which typically rely on clinical evaluation and radiographic imaging. The Lauge-Hansen classification is one of the most widely used systems (2). It is based on cadaver studies, where fresh cadaver legs were fixed using a vise and nails, and rotational forces (abduction, adduction, or external rotation) were applied while the foot was in maximum supination or pronation. Supination of the foot consists of a composite motion including internal rotation, adduction (hindfoot), and inversion (forefoot). Pronation includes external rotation, abduction (hindfoot), and eversion (forefoot). Lauge-Hansen established the ankle fracture classification system through multiple experiments, based on the foot position and the direction of the injuring force, dividing fractures into four categories: supination-external rotation, supination-adduction, pronation-external rotation, and pronation-abduction types (1, 2, 9, 10). Moreover, the system suggests that fracture injuries occur in a predictable sequence, with no stages skipped (11, 12).

When the foot is in maximal supination, the adduction of the talus compresses the medial malleolus. As the lateral ligaments of the ankle joint become taut, the foot becomes a rigid unit. If a forceful adduction is applied at this point, it exerts a strong pull on the lateral collateral ligaments while placing abnormal pressure on both medial and lateral malleoli, leading to a SAD fracture (1, 9). This type of fracture occurs in two stages: In the first stage, a transverse fracture of the lateral malleolus below the level of the tibial articular surface occurs, or there is a rupture of the lateral ankle ligaments. In the second stage, a vertical fracture of the medial malleolus occurs (1, 2). According to the Lauge-Hansen classification, SAD fractures account for approximately 12.2% of all ankle fractures (6).

Arimoto and Forrester developed an algorithm based on the Lauge-Hansen classification to interpret radiographic images. This algorithm helps identify injury categories, recognize fracture types, and predict ligament injuries, making clinical application more practical. The method involves these steps: first, identify if a distal fibular fracture is present and determine the fracture height and type (e.g., oblique, transverse, spiral). If a fibular fracture exists, further evaluation is needed for additional injuries, including posterior malleolus fracture, medial malleolus fracture, tibiofibular clear space, and distal tibial joint space. If there is no fibular fracture, assess for a medial malleolus fracture or medial clear space widening (9, 13). Following this algorithm is crucial for accurate injury classification (9). According to the Arimoto and Forrester algorithm, the presence of a transverse fracture of the distal fibula below the tibial plafond, along with a medial malleolus fracture, defines a SAD stage 2 injury. If no fibular fracture is observed but a vertical fracture of the medial malleolus is present, it can still be classified as SAD stage 2. This typically implies a rupture of the ATFL or calcaneofibular ligament, evidenced by widening of the lateral joint space.

The ankle joint is a complex structure composed of bones and ligaments. Its stability can be conceptualized as two osseofibrous rings: the axial ring (composed of the lateral malleolus, anterior inferior tibiofibular ligament, medial malleolus, and posterior inferior tibiofibular ligament) and the coronal ring (composed of the lateral malleolus, lateral ligaments, talus, deltoid ligament, and medial malleolus) (14, 15). When the ankle joint is subjected to trauma, the injuring force disperses along different paths depending on the mechanism of injury, resulting in sequential and predictable damage to the bone and ligament structures within the osseofibrous rings (14). The failure point of this bone-ligament-bone system usually occurs at the position of least resistance, depending on the quality of the bones and ligaments. This can manifest as fractures in areas of lower bone density, osseous avulsions of ligaments, or simple ligament tears (16–18). Influenced by these theories, based on the preoperative radiographic images, we believe the patient fits the injury pattern of a SAD fracture. Clinically, it is generally considered that when a lateral malleolus fracture is present, associated ligament rupture at the lateral malleolus is unlikely. As described by the algorithm, when a transverse fibular fracture and a medial malleolus fracture are present, the evaluation does not include checking for lateral ligament ruptures. This is because the injuring force acting on the coronal ring typically dissipates with a lateral malleolus fracture, which is the point of least resistance.

However, during surgery, we encountered a unique situation. Despite successful reduction and fixation of the medial and lateral malleoli, multiple C-arm fluoroscopy views revealed that the talus could not be repositioned and remained internally rotated, which immediately drew our attention. Physical examination revealed a positive anterior drawer test. Considering the patient's history, physical findings, imaging, fracture classification, and the functional characteristics of the ligaments, we suspected an ATFL injury. After extending the lateral incision and exploring the area, we found that the ATFL was completely ruptured, while the calcaneofibular ligament remained intact. Intraoperatively, it was clearly visible that there was a complete discontinuity of the ATFL with only a few ineffective connective fibers remaining, indicating a loss of structural integrity. The ATFL originates at the anterior margin of the lateral malleolus and runs anteromedially to insert on the talar body (19, 20). It is the most frequently injured ligament in the lateral ligament complex (19, 21–23). Its primary role is to prevent anterior displacement of the talus in the sagittal plane and to restrict inversion and internal rotation of the talus on the tibial axis (21, 22, 24). This was confirmed after we repaired the ligament, as subsequent C-arm fluoroscopy showed good alignment.

Gardner et al. (25) conducted an MRI study to assess the ability of the Lauge-Hansen classification to predict ligament injury and its mechanisms in ankle fractures. By comparing the Lauge-Hansen classification's predictions with MRI-observed ligament injuries, the study found that in 59 cases of ankle fractures, over 65% of patients had complete ligamentous injury alongside fractures at the ligament attachment site. However, Warner et al. (26) challenged these results. They evaluated ligament injuries using the Lauge-Hansen classification, MRI, and combined this with intraoperative physical examination and direct exploration. Among the 283 patients studied by Warner et al., they did not find instances of complete ligament tears occurring simultaneously with ankle fractures at the attachment site, contradicting Gardner et al.'s findings. Warner suggested that Gardner et al. may have overestimated complete ligament tears by focusing on MRI features like interstitial edema and wavy or curled fibers. Okanobo et al. (9) described a simplified approach to the Lauge-Hansen classification. They noted that SAD stage 1 typically presents as a transverse fracture of the lateral malleolus at or below the level of the ankle joint, accompanied by an ATFL rupture or calcaneofibular ligament tear. However, they did not provide specific evidence of concurrent transverse fibular fractures and ATFL ruptures. In contrast, more reviews of the Lauge-Hansen classification did not report this occurrence in SAD stage 1 (1, 2, 10, 16). In our study, we found lateral injuries in SAD fractures that included both distal transverse fibular fractures and ATFL ruptures, aligning with the description by Okanobo et al. for SAD stage 1.

Ankle fractures are essentially bone-ligament injuries affecting the two osseofibrous rings. According to the Lauge-Hansen theory, pure ligament injuries without bone involvement and ligamentous avulsions manifesting as fractures are referred to as soft tissue equivalents of fractures (16). Taking the ATFL as an example, this ligament injury commonly occurs during lateral ankle sprains (19). If not properly treated after the initial injury, there's a heightened risk of developing chronic ankle instability (19, 21, 24, 27). The primary goal of treating ankle fractures is to restore the congruity of the tibiotalar joint and ensure joint stability (14). In this case, the rupture of the ATFL directly affected the repositioning of the talus. If the injury were not identified intraoperatively, it might lead to recurrent pain, chronic ankle instability, and potentially arthritis. Therefore, assessing ligament injuries in the ankle is crucial for restoring ankle stability (14, 28, 29). Especially during surgery, if a hidden ligament injury is suspected, timely physical examination or open exploration is necessary to evaluate ankle stability.

To verify the rarity of this specific case, we conducted a case collection study, reviewing fresh ankle fracture cases from the past year in our department. We identified 17 cases of SAD fractures: 10 were stage 1, and 7 were stage 2. Among the 10 stage 1 cases, 5 presented with transverse fractures of the lateral malleolus, while the other 5 showed lateral ligamentous avulsions. In all 7 stage 2 cases, there were vertical fractures of the medial malleolus; 5 had transverse lateral malleolus fractures, and 2 exhibited lateral ligament injuries. For patients with transverse fractures of the lateral malleolus, we reviewed surgical records to determine if anchors were used to repair the lateral ligaments. Results showed that in all 10 cases with transverse lateral malleolar fractures, there were no findings of lateral ligament ruptures. However, intraoperative evaluation of the ATFL integrity might not have been routinely performed, possibly affecting the reliability of this data. Regarding the injury mechanism of this patient, we hypothesize that during the sprain, the foot was in a supination position and subjected to an inversion force, leading to the initial rupture of the ATFL. As the patient lost balance, the continued inversion force eventually caused the distal fibular fracture. Additionally, the patient's obese status (BMI: 30.4 kg/m^2^) might have contributed to the occurrence of this unique injury combination, where both a transverse lateral malleolar fracture and ligament rupture were present.

This case report has certain limitations. Initially, no preoperative MRI was performed because the patient's DR images aligned with the Lauge-Hansen SAD stage 2 criteria, suggesting, per Lauge-Hansen classification, that the ATFL should be intact in the presence of a transverse lateral malleolus fracture. However, during surgery, using fluoroscopy to assess the ankle alignment and stability, we promptly explored and repaired the ruptured ATFL, achieving a satisfactory reduction result. Secondly, we could not establish a case series, as no similar injury patterns were found among SAD fracture cases in the past year. Therefore, future studies should include more cases and intraoperative exploration to evaluate the potential presence of occult ATFL injuries alongside transverse lateral malleolus fractures in SAD injuries.

## Conclusion

In summary, we identified a case of SAD ankle fracture where a transverse fracture of the lateral malleolus was accompanied by a rupture of the ATFL. This combination of injuries is relatively rare and may be related to the intensity of the force or individual anatomical variations. It is often overlooked in clinical assessments. By sharing and studying such cases, awareness of these occult injuries can be increased, reminding clinicians that in SAD stage 2 fractures with unexplained talar tilt after fixation they should suspect and evaluate for occult ATFL injury, thereby formulating more effective rehabilitation plans.

## Data Availability

The original contributions presented in the study are included in the article/Supplementary Material, further inquiries can be directed to the corresponding author.
